# Could gastrointestinal disorders differ in two close but divergent social environments?

**DOI:** 10.1186/1476-072X-11-5

**Published:** 2012-02-06

**Authors:** Ewa Grodzinsky, Claes Hallert, Tomas Faresjö, Elisabet Bergfors, Åshild Olsen Faresjö

**Affiliations:** 1 R & D Unit, Local Health Care, County Council of Östergötland, SE-581 85 Linköping University, Linköping, Sweden; 2 Dept of Social and Welfare Studies, Campus Norrköping, Linköping University, SE-581 83 Linköping, Sweden; 3 Dept of Medicine and Health Sciences, Linköping University, SE-581 83 Linköping, Sweden

**Keywords:** Social environment, General population, Gastrointestinal disorders, Sex, Public health

## Abstract

**Background:**

Many public health problems in modern society affect the gastrointestinal area. Knowledge of the disease occurrence in populations is better understood if viewed in a psychosocial context including indicators of the social environment where people spend their lives. The general aim of this study was to estimate the occurrence in the population and between sexes of common gastrointestinal conditions in two neighborhood cities representing two different social environments defined as a "white-collar" and a "blue-collar" city.

**Methods:**

We conducted a retrospective register study using data of diagnosed gastrointestinal disorders (cumulative incidence rates) derived from an administrative health care register based on medical records assigned by the physicians at hospitals and primary care.

**Results:**

Functional gastrointestinal diseases and peptic ulcers were more frequent in the white-collar city, while diagnoses in the gallbladder area were significantly more frequent in the blue-collar city. Functional dyspepsia, irritable bowel syndrome, and unspecified functional bowel diseases, and celiac disease, were more frequent among women while esophageal reflux, peptic ulcers, gastric and rectal cancers were more frequent among men regardless of social environment.

**Conclusions:**

Knowledge of the occurrence of gastrointestinal problems in populations is better understood if viewed in a context were the social environment is included. Indicators of the social environment should therefore also be considered in future studies of the occurrence of gastrointestinal problems.

## Background

Many public health problems in modern society affect the gastrointestinal area. Knowledge of the disease occurrence in populations is better understood if viewed in a psychosocial context including indicators of the social environment where people spend their lives. The etiology of functional gastrointestinal diseases (FGD) such as irritable bowel syndrome (IBS), functional dyspepsia (FD) and gastroesophageal reflux (GERD) is complex and in many ways still unclear and there is still a lack of understanding of the pathology [1,2]. The dysfunctions that lead to or aggravate FGD are a combination of biological, psychological and psychosocial factors [3]. These multiple sets of factors are likely to interact in the pathogenesis and clinical manifestations of the disorders. This multi-factorial pattern is often evident in other gastrointestinal disorders and in many other public health diseases in a complex contemporary society. Several attempts have been made to create bio-psycho-social models to help bring some order to factors and to explain the interaction of environmental, cultural, social, psychological and biological factors [4-6].

Knowledge of the disease prevalence rates is crucial for describing the population burden of diseases and assessing the associated health care utilization and health care cost [7]. Available sources for this purpose include health surveys, screening investigations, and register studies [8]. Previous studies of functional gastrointestinal disorders such as IBS have shown the value of using administrative health care registers [9,10]. The General Practice Research Database (GPRD) in United Kingdom (UK) had also been used in different studies of several diseases such as GERD and chronic obstructive pulmonary disease [11,12] as well as detection of colorectal tumor and inflammatory bowel disease (IBD) among patients with IBS [13,14].

The role and importance of social and physical environments for health, the effect of residence and the question of whether we should focus on context rather than people is still an open question in health sciences [15-17]. A general conclusion as regards this issue is that who you are, but also how and where you live your life are both of importance for health outcomes [18]. In order to address this issue, we conducted a study focusing on the population in two equally sized Swedish cities, so called the "Twin cities". These two cities are geographically close and part of the same public health care system managed by Östergötland County Council. However, the social and cultural history and current social structure of these two cities differ; one city could be described as a "blue-collar" city and the other as a "white-collar" city. Thereby, these twin cities are neighborhood cities but represent two different social environments.

### Study aims

The general aim of this study was to estimate the occurrence in the population and between sexes of common gastrointestinal conditions in different social environments defined as a "white-collar" and a "blue-collar" city.

## Methods

### The study population in the twin cities

We focused on the population in two equally sized cities (the twin cities) located 40 km (25 miles) apart in the same county in the south-east of Sweden, namely, the city of Norrköping (N) and the city of Linköping (L). The two cities are quite equal in size and there are neither any age nor sex distribution differences between them [19]. Both cities are managed by the same health care organization; the county council is responsible for all public-funded health care in the two cities. However, the past and even the current socio-economic structures in the two cities are different, see Table 1. One of the cities (L) is dominated by high-tech industries and higher education institutions, and may be regarded as a white-collar city, while the other (N) has a long industrial history and could be viewed upon as a blue-collar city. Although the cities today have an extrinsic resemblance in terms of physical environment and climate, public health is remarkably different in these two cities [19]. This is manifested in a number of public health indicators including life expectancy, prevalence of ischemic heart disease, sick leave, and lifestyle factors consistently in favour of the "white-collar" city [19,20].

**Table 1:** Indicators of the social environments in the "white-collar" and "blue-collar" twin city

		White-collar twin city	Blue-collar twin city
Total number of inhabitants (2009)		144 690	129 254
Life expectancy at birth	Women	82.6	81.8
	Men	79.5	77.9
Inhabitants born outside Europe (%)		9.6	11.0
Inhabitants aged 20-64 years with high income (%) *		21.2	16.9
Inhabitants aged 20-64 years with high education (%) **		45.9	30.7
Mean yearly total income per inhabitant over 16 years (SEK)		209 000	197 000
Economic social aid per inhabitant in the city (SEK)		1 391	1 715
Unemployment rate aged 20-64 years (%)	Women	3.0	4.0
	Men	4.3	5.5
Crime of violence, number per 100 000 inhabitants		976	1 506
Participation in National elections (%)		85.0	81.5

All data valid for 2006 and from Official Statistics, the Swedish Institute of Public Health and Statistics, Sweden

*High income refers to the 20% of the inhabitants nationally with the highest income. **High education refers to 12 or more years of education

### Data coding

There is a long tradition in the Scandinavian countries of documenting diseases in registers and the integrated health care delivery systems are obligated to serve all inhabitants, and each inhabitant is assigned a unique personal code number based on birth date and sex [21]. The health care database can offer data also suitable for population-based epidemiological studies. The county councils in Sweden are required by law to report inpatient data to the Swedish Hospital Discharge register on an annual basis, but national registration of outpatient and primary care data has so far not been implemented [21]. However, in one region and county council in Sweden (Östergötland County Council), patient data from Primary Health Care (PHC), outpatient and, inpatient care (hospital patients) have been recorded for some years in a complete computerized population-based Health Care Register (HCR).

We conducted a retrospective register study using a health care register to identify diagnosed gastrointestinal problems in medical records. The Health Care Register (HCR) used is based on computerized data files linked by a unique personal code to birth date and sex of all inhabitants in the county. The same personal code is used for all visits and diagnoses in the HCR. The international classification of diseases (ICD) 10-code was used. The selected GI disorders in this study were: Irritable bowel syndrome-constipation (IBS-C) and Irritable bowel syndrome-diarrhoea (IBS-D), respectively (IBS; ICD-K58.0-K58.9), esophageal reflux (GERD; ICD-K21), functional dyspepsia (FD; ICD-K30), functional bowel disease unspecified (FGD-UNS; ICD-K59.0-K59.9), peptic ulcer disease (PUD; ICD-K25-K26), inflammatory bowel disease (IBD; ICD-K50-K51), coeliac disease (ICD-K90) diseases in the gallbladder area (ICD-K80-K83.9), cancers in the GI tract (ICD-C16, C17, C18 and C20) and alcohol-related liver diseases (ARLD; ICD-K70). The attending physician assigned all diagnostic codes. The diagnoses were extracted from the HCR using a case-finding algorithm that retrospectively searched the register from 1/1/2002 to 31/12/2007.

### Data analysis

All data were stored in a common database and statistically analysed using the SPSS version 17.0 program (SPSS Inc., Chicago, IL, USA). The algorithm captured the cases (one case = one patient) regardless of whether the disorders of interest constituted the main or secondary diagnoses, and it also specified the health care level at which the patient was diagnosed, that is, primary care, outpatient hospital care, and/or inpatients hospital care. The following case definition was applied: the first contact with health care services with a GI diagnosis during the period studied (2002-2007) was regarded as a case. Cumulative incidence rates were calculated and expressed as number of cases per 1,000 inhabitants. The mean population in different age groups for the period 2002-2007 was used as the denominator. We initially estimated the occurrence of gastrointestinal diagnoses for all ages up to the age of 80 in a gender comparison. However, the comparison between the blue-collar and the white-collar cities were only focusing on the population between 25 and 79 years, i.e. the working ages and retirement ages. The numerator for the prevalence calculation was the number of first diagnosed cases identified during the six-year study period. The denominator was the number of inhabitants calculated as the mean number in each age group of the population for the six-year study period. The prevalence rates are presented as numbers per 1,000 inhabitants. We also calculated the Relative Rates (RR) and 95% confidence intervals (CI) and differences between the cities were also calculated with Pearson's chi square.

The Research Ethics Committee of the Faculty of Health Sciences, Linköping University, Sweden, approved the study (2009).

## Results

### Comparison between the social environments

The comparison between the twin cities revealed significant differences for functional GI-disorders and peptic ulcer disease (PUD) (ICD-K25 to ICD-K26) that were more frequent in the "white-collar" city than in the "blue-collar city. On the other hand, the diagnoses in the gallbladder area (ICD-K80.0 to ICD-K83.9) were significantly more frequent in the blue-collar city for both sexes and in all age groups. Also alcohol-related liver disease (ARLD) (ICD-K70) among 45-64 year old patients of both sexes was more frequent in the "blue-collar" city; see Table 2 and Table 3.

**Table 2:** Cumulative incidence of GI-diagnoses for males in the "white-collar" and the "blue-collar" city (the Twin cities) in the age-groups 25-79 years, numbers per 1,000 inhabitants

	White-collar twin city (L)			Blue-collar twin city (N)		
**GI diagnoses** **Functional diseases**	**25-44 years**	**45-64 years**	**65-79 years**	**25-44 years**	**45-64 years**	**65-79 years**
	
		**RR (95% CI)**			**RR (95% CI)**	

GERD Esophageal reflux	10.8 ***	20.2 ***	29.0 ***	5.6	8.0	9.5
	
	1.95 (1.53-2.47)	2.51 (2.05-3.07)	3.01 (2.27-3-97)	1.0	1.0	1.0

FD Functional dyspepsia	10.2**	13.1***	16.0**	7.0	7.3	8.6
	
	1.42 (1.17-1.82)	1.80 (1.44-2.25)	1.84 (1.34-2.52)	1.0	1.0	1.0

IBS-D, Irritable Bowel Syndrome - Diarrhoea	2.0*	2.1*	1.4	1.1	1.0	1.0
	
	1.76 (1.00-3.07)	2.00 (1.12-3.57)	1.35 (0.88-6.88)	1.0	1.0	1.0

IBS-C, Irritable Bowel Syndrome - constipation	3.0*	3.0*	2.0	2.0	2.0	2.0
	
	1.64 (1.05-2.56)	1.56 (1.00-1.46)	1.0	1.00	1.00	nt

FGD-UNS Functional Bowel Disease unspecified	5.9	10.0	26.0	7.0	10.1	33.0*
	
	1.00	1.00	1.00	1.17 (0.91-1.51)	1.01 (0.90-1.12)	1.24 (1.02-1.50)

PUD, Stomach and Duodenum ulcer	3.5**	11.0***	30.0***	1.8	5.7	15.4
	
	2.00 (1.31-3.06)	1.92 (1.49-2.47)	1.95 (1.55-2.47)	1.00	1.00	1.00

Gallstone, gallbladder inflammation and other diseases in the gallbladder area	9.2	18.4	37.0	11.5*	22.2*	48.0*
	
	1.00	1.00	1.00	1.24 (1.02-1.51)	1.21 (1.04-1.41)	1.29 (1.10-1.52)

Alcohol-related liver diseases	0.5	2.4	4.2	1.1	4.6**	2.4
	
	nt	1.00	1.76 (0.96-3.23)	nt	1.88 (1.27-2.76)	1.00

***p < 0.0001, **p < 0.001 *p < 0.05 nt = not tested

**Table 3:** Cumulative incidence of GI-diagnoses for females in the "white-collar" and the "blue-collar" city (the Twin cities) in the age-groups 25-79 years, numbers per 1,000 inhabitants

	White-collar twin city (L)			Blue-collar twin city (N)		
**GI diagnoses** **Functional diseases**	**25-44 years**	**45-64 years**	**65-79 years**	**25-44 years**	**45-64 years**	**65-79 years**
	
		**RR (95% CI)**			**RR (95% CI)**	

GERD Esophageal reflux	9.1***	20.6***	29.0 ***	3.6	8.7	9.2
	
	2.53 (1.88-3.40)	2.51 (2.05-3.07)	3.02 (2.32-3-91)	1.00	1.00	1.00

FD Functional dyspepsia	18.0***	24.0***	24.0***	8.2	8.0	10.2
	
	2.18 (1.76-2.66)	3.04 (2.49-3.72)	2.34 (1.81-3.02)	1.00	1.00	1.00

IBS-D, Irritable Bowel Syndrome - Diarrhoea	7.0***	5.5***	5.0 **	2.6	2.4	1.6
	
	2.62 (1.86-3.71)	2.28 (1.57-3.33)	3.03 (1.62-5.65)	1.00	1.00	1.00

IBS-C, Irritable Bowel Syndrome - constipation	7.0*	7.0	6.5	5.0	6.0	4.2
	
	1.41 (1.07-1.87)	1.15 (0.87-1.50	1.28 (0.83-1.97)	1.00	1.00	1.00

FGD-UNS Functional Bowel Disease unspecified	18.0**	19.0*	32.0	13.0	16.2	32.0
	
	1.38 (1.16-1.64)	1.18 (1.00-1.39)	nt	1.00	1.00	nt

PUD, Stomach and Duodenum ulcer	3.0**	7.5***	24.0***	1.0	4.0	8.0
	
	2.61 (1.51-4.53)	1.87 (1.39-2.54)	2.97 (2.24-3.93)	1.00	1.00	1.00

Gallstone, gallbladder inflammation and other diseases in the gallbladder area	25.6	32.0	35.0	35.0***	44.0***	47.0**
	
	1.00	1.00	1.00	1.36 (1.21-1.53)	1.37 (1.22-1.53)	1.35 (1.16-1.57)

Alcohol-related liver diseases	0.3	0.8	1.3	0.2	1.8*	0.9
	
	nt	1.00	1.35 (0.54-3.35)	nt	2.23 (1.16-4.28)	1.00

***p<0.0001, **p<0.001 *p<0.05 nt = not tested

### Sex differences

**Figure 1:**
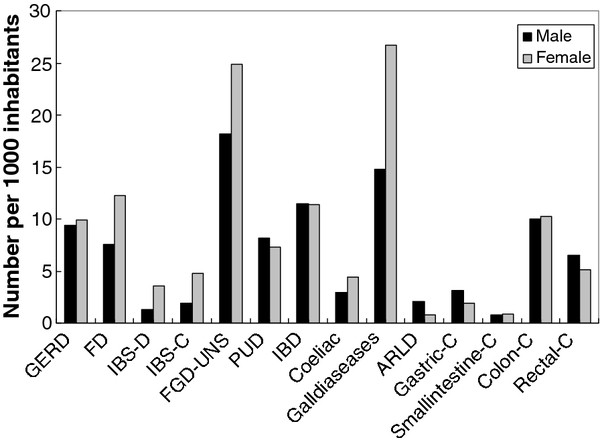
**Cumulative incidence of GI diagnoses (per 1.000 inhabitants) for females and males in a defined region**. (GERD = Esophageal Reflux, FD = Functional Dyspepsia, IBS-C = Irritable Bowel Syndrome--constipation, IBS-D = Irritable Bowel Syndrome--diarrhea, FGD-UNS = Functional Bowel Disease unspecified, PUD = Peptic Ulcer Disease, IBD = Inflammatory Bowel Disease, ARLD = Alcohol Related Liver Disease, C = cancer.).

Significantly differences between sexes in the total cumulative incidence rates of different gastrointestinal disorders were also seen, the same pattern concerning sex differences were seen in both cities, the total distribution of these differences are illustrated in Figure 1. Women had significantly more FD diagnoses than men, RR = 1.60 (95% CI 1.48-1.73). This trend was also seen for IBS-C, RR = 2.55 (95% CI 2.20-2.95) and IBS-D, RR = 2.61 (95% CI 2.29-3.11) except for the lowest age group, where men tend to have more IBS diagnoses. For unspecified FGD, more diagnoses were seen among women RR = 1.37 (95% CI 1.30-1.45). Significantly more diagnoses of GERD were seen among men in the age groups 25-44 RR = 1.29 (95% CI 1.08-1.52) and 80 + RR = 1.71 (95% CI 1.21-2.28) but not totally. Even peptic ulcer disease were more frequent among men compared to women in the age groups from 25 and older, RR = 1.12 (95% CI 1.02-1.23).

Coeliac diagnoses were significantly, RR = 1.57 (95% CI 1.37-1.79), more frequent among women compared to males as well as diagnoses of the gallbladder area, RR = 1.80 (95% CI 1.71-1.91). Even ARLD showed a sex difference, more frequent among men, RR = 2.69 (95% CI 2.05-3.53). Gastric (ICD-C16) and rectal cancers (ICD-C20) were totally more frequent among men, RR = 1.73 (95% CI 1.34-2.21) and RR = 1.27 (95% CI 1.08-1.48), respectively, as illustrated in Figure 1.

## Discussion

### Main findings

This population-based study revealed differences in the occurrence of GI diagnoses between the studied social environments and also between sexes regardless of the social environment. The comparisons showed that GI-diagnoses that are predominantly related to stressful life events like GERD, IBS, and PUD were more common in the population of the "white-collar" city. Possibly this difference in type of gastrointestinal occurrence between these social environments reflects differences in perceived and actual exposure to stressful life conditions, more predominant in the "white-collar" city. Recent studies of functional gastrointestinal disorders such as IBS, showed that e.g. lack of influence on planning work, anxiety and sleep disruptions were associated with an IBS diagnosis in women and lack of influence on working pace among men [22,23]. Although this study was only made in the "white-collar "city these attributes were more frequent among the "blue-collar" population in that specific city. These factors might also lead to a stressful life situation that might affect the GI area. PUD and GERD might also, among other things, be induced by stressful life conditions. Another putative candidate mechanism that might explain the differences between the two social environments is a relationship between socioeconomic status and psychiatric disorders on the one hand and psychiatric disorders and FGD on the other hand [3,22,23].

However, certain GI diagnoses like alcohol-related liver diseases and gall-tract disorders were more prevalent in the "blue-collar" city. This may suggest a less healthy life style in its population leading to these problems. This is supported by our previous observations showing that diagnoses predominantly related to life-style problems like coronary heart diseases and chronic obstructive pulmonary disease were more common in the studied blue-collar city [20].

### Discussion under the light of literature

The observed differences in the occurrence of GI diagnoses between the two social environments are not likely to be explained by chance or by differences in access to health care services. However, it could not be ruled out that health care seeking patterns might differ in the two cities. Both the inpatient and outpatient care is delivered by public health services based on population needs in both cities. So there are no significant differences in the supply of health care facilities like primary care in the two cities. On the marginal, the "white-collar" city population might benefit from being more often referred for investigations requiring technology available only at a university hospital, since these facilities is situated in the "white-collar" city. There might also be variations between the cities in the diffusion of diagnostic skills of doctors within the field of GI disorders. This might impact the assignment of GI complaints in patients seeking advice in the primary care. Anyhow, we believe that the differences seen between the population in the two cities is related to differences in health behaviors, educational levels, psychosocial factors, nutritional habits, life styles in general and specifically alcohol consumption and smoking behaviors [19].

It is well known that FGD diseases such as IBS and FD and FGD-UNS are more common among women than men, but surprisingly, IBS and FGD-UNS were more frequent among the youngest group of men (data not shown). The explanations of this gender phenomenon must be hypothetic and can differ. It might be randomly due to an unknown factor or due to the fact that more women suffer from IBS [24-26] and therefore seek health care more often when their children having the same GI complaints. Our recent study showed that a family history of IBS was related to an IBS diagnosis for both sexes [23]. One hypothesis might be that young men are more vulnerable to psychosocial stressful conditions in everyday life than young women, or quite simply, they have been eating too much, too little or are allergic to certain kinds of food, which might explain the higher frequency of FGD-UNS among boys. The prevalence of GERD was fairly equal between men and women. Alcohol-related liver disease was more frequent among men, since men in general consume more alcohol than women. Coeliac diagnoses were significantly more frequent among women compared to men and most frequent among younger populations. Specially, adult women with coeliac disease continue to seek health care for other complaints, without mentioning their previous coeliac diagnose [27].

The total number of gall diseases was significantly more frequent among women compared to men except in the oldest age group. The causes of gallstone formation are multifactorial, although some risk factors are permanent, such as being women, increasing age and ethnicity and previous family history of the disease. Other recognized risk factors are a high caloric intake and obesity [28-30]. There are two types of gallstones. In the western world, the majority of the stones consist predominantly of cholesterol crystals (80-85%) and the rest black pigment [29]. Brown pigment stones, however, consist of calcium bilirubinate, calcium soaps, mucin and some cholesterol and are the predominant type in East Asia [31]. However, gallstone composition in East Asia has changed in recent decades with an increase in cholesterol gallstones possibly due to changed dietary habits [32]. Studies also show that gallstones are uncommon in children [33]. The risk of getting gallstones increases with age in all ethnic groups [34-36]. Women in the USA and Western Europe compared to Asia have a larger risk of this disease most likely due to the composition of the gallstones [37-39]. Female hormones as well as increasing obesity for women might explain some of the sex differences found were females are more at risks for gallstones than men [40,41]. However, this phenomenon diminishes after menopause, which our study also shows, since the frequency is more equal among sexes in the two oldest age groups.

Peptic ulcer diseases in this study occur more frequently among men in all age groups. These diseases are often induced by Helicobacter pylori (HP) or non-steroid anti-inflammatory drugs (NSAID), two independent risk factors, PUD is often decreasing with age, although a peak often occur at retirement [42,43]. However, these factors cannot explain differences in occurrence between sexes. One hypothesis might be that men to a larger extent are more stressed and this induces the HP bacteria to act and induce PUD, or men might to a greater extent with increasing age suffer from complaints like arthritis and musculoskeletal problems that require higher consumption of NSAID and acetylsalicylic acid (ASA).

This study also showed that gastric and rectal cancers occurred more frequently among men of all ages compared to women. Colon and small intestinal cancers were more equally distributed among both sexes, although colon cancer was slightly more frequent among men, but not significantly so. Colorectal cancer is the third most common form of cancer in Sweden [44]. On the other hand, it is also the third leading cause of death in the USA [45]. Studies suggest that there might be sex differences in screening for colorectal cancers, i.e. that physicians may refer men more frequently for screening than women [46]. Women might report greater fear, anxiety, and embarrassment about screening procedures, which could affect the behaviour concerning this kind of screening [47-49]. These factors might explain sex differences, but to what extent? Screening for colorectal cancer is recommended in the USA as well as in some countries in Europe, although not in Sweden so far [50-52]. However, the etiology of all these GI diseases is beyond the scope of this study.

### Study limitations and strengths

Possible limitation in this study is that we do not have any measures of individual socio-economic conditions. Living in a certain social environment like a "blue-collar" or a "white-collar" city could only be a proxy for real socioeconomic status. Further limitations in the study are that the data used was not initially collected for research purposes. The diagnoses registered, although they all derive from medical records, could in some cases be regarded as approximate. The strength of this study is that it is based on medical records in computerized data files (HCR) for all inhabitants linked by birth date and sex. The same personal code is used for all visits and diagnoses in HCR. An individual can thus be followed retrospectively or prospectively through the health care system using this personal code. The health care institution where the patient was diagnosed represents all health care levels - primary care, outpatient hospital care, and/or inpatient hospital care which gives a more complete panorama of the health care. Other strengths of this study are that our inclusion method gains reliability through being very general and covering a span of several years and that the number of included patients is high tending to level out possible misclassification within the groups defined as patients. Studies have also shown that general practitioners rarely misdiagnose for instance FGD [53-55] in fact, there may be a tendency by the GP's not to set FGD diagnosis such as IBS in primary care. However, a study of the quality and content of the Swedish Hospital Discharge Register indicates 95% coverage of main diagnostic codes in inpatient care in this region in 1986 and 98% in 2002 [56]. Validation of HCR and other administrative data has shown high specificity in registers covering all types of health care [21,57-59]. But a constraint for register data is that misclassifications do occur, including cases that are not recorded because they are overlooked or given incorrect clinical codes. ICD code registration could vary between physicians and health care centers and also between diagnoses [60]. Nevertheless it can be assumed that the data used in this study based on Health Care register (HCR) has high reliability.

### Impact and conclusions

To conclude, this population-based study revealed differences in the occurrence of gastrointestinal diagnoses between the populations in a "white-collar" and a "blue-collar" city. Gastrointestinal diagnoses that are predominantly related to stressful life events like GERD, IBS and peptic ulcer disease was more common in the population of the "white-collar" city. This might reflect differences in lifestyles, living conditions and factors in working life like differences in heavy, strenuous or even monotonous work of the populations in the two studied cities that constitutes two close but different social environments. Lifestyle-related gastrointestinal diseases like alcohol-related liver diseases and gall-tract disorders were more frequent in the "blue-collar" city. Significant differences in the cumulative incidence rates of the most common gastrointestinal conditions between sexes were also seen. Women tended to have more functional gastrointestinal diagnoses and gall diagnoses, while men had more peptic ulcer diseases and alcohol-related liver diseases. Many of these conditions also tend to increase with age. A forthcoming research project will investigate the validity of diagnose-issue and whether differences in working life conditions between individuals with functional gastrointestinal problems compared to healthy controls in the "white-collar" and the "blue-collar" city might contribute to explain the differences seen in these twin cities.

Knowledge of the occurrence of gastrointestinal problems in populations is better understood if viewed in a context were the social environment is included. Indicators of the social environment should therefore also be considered in future studies of the occurrence of gastrointestinal problems.

## Abbreviations

ARLD: Alcohol-related liver disease; ASA: Acetylsalicylic acid; CI: Confidence interval; FD: Functional dyspepsia; FGD: Functional gastrointestinal diseases; GERD: Gastroesophageal reflux; GI: Gastrointestinal; GPRD: The general practice research database; HCR: Population based health care register; HP: Helicobacter pylori; IBD: Inflammatory bowel disease; IBS: Irritable bowel syndrome; IBS: C Irritable bowel syndrome constipation; IBS: D Irritable bowel syndrome diarrhoea; ICD: International classification of diseases; L: Linköping; N: Norrkoping; NASID: Non-steroid anti-inflammatory drugs; PHC: Primary health care; PUD: Peptic ulcer disease; RR: Relative risks; UK: United Kingdom; UNS: Unspecific.

## Competing interests

The authors declare that they have no competing interests.

## Authors' contributions

EG, ÅF, TF participated in the study design and coordination and completed the data collection. EG, ÅF, TF, CH and EB drafted the manuscript as well as analysis and interpretation of data, read and approved the final manuscript.
